# Exercise Obsession and Compulsion in Adults With Longstanding Eating Disorders: Validation of the Norwegian Version of the Compulsive Exercise Test

**DOI:** 10.3389/fpsyg.2019.02370

**Published:** 2019-10-22

**Authors:** Karianne Vrabel, Solfrid Bratland-Sanda

**Affiliations:** ^1^Research Institute, Modum Bad Psychiatric Center, Vikersund, Norway; ^2^Department of Sports, Physical Education and Outdoor Life, University of South-Eastern Norway, Kongsberg, Norway

**Keywords:** methods, anorexia nervosa, bulimia nervosa, eating disorder not otherwise specified, self-report measures, adults

## Abstract

**Objectives:**

The objectives of this study were to (1) validate the Norwegian version of the Compulsive Exercise Test (CET) in adults with longstanding eating disorders, and (2) explore predictors of high CET-score.

**Methods:**

Adult inpatients (*n* = 166) with longstanding DSM-IV Anorexia Nervosa, Bulimia Nervosa (BN) or Eating Disorder not Otherwise Specified (EDNOS) completed the CET instrument, Eating Disorder examination questionnaire (EDE-Q), Beck Depression Inventory-II (BDI-II) and Symptom checklist-90 (SCL-90). A total CET score of 15 or above was defined as high CET-score. ANOVA, Confirmatory factor analysis, Pearson’s correlation, and logistic regression were used to analyze the data.

**Results:**

Cronbach’s alpha varied from 0.68 to 0.96 for the CET and its subscales. The confirmatory factor analysis showed adequate fit. Convergent validity of the CET demonstrated correlation between EDE-Q global and subscale scores and CET total score. The same pattern was found for correlation between CET subscales and EDE-Q subscales. EDE-Q global score and frequency of exercise episodes predicted high CET-score, yet 21% of the patients with high CET score had less than one episode of exercise per week.

**Conclusion:**

The Norwegian version of CET is valid and useful for assessing compulsive exercise in a sample with longstanding ED. The understanding of compulsive exercise must to a greater extent differ between obsessions and compulsions, as a significant number of patients with high CET score showed no or little exercise behavior.

## Introduction

Compulsive exercise has been shown to be one of the core symptoms of ED in general and AN in particular, and this symptom is associated with more severe psychopathology, poorer treatment outcome and higher risk of relapse (Strober et al., 1997; Shroff et al., 2006; Meyer et al., 2011). Although there is a lack of consensus on how to conceptualize compulsive exercise, some studies have suggested a multifactorial etiology for compulsive exercise (Meyer and Taranis, 2011; Meyer et al., 2011). Based on a cognitive behavioral model, Meyer et al. (2011) proposed four key constructs underlying compulsive exercising: (1) eating psychopathology; (2) obsessive compulsiveness; (3) mood regulation; and (4) perfectionism. This multidimensional definition of compulsive exercise led to the development of a new self-report instrument, the Compulsive Exercise Test (CET) (Taranis et al., 2011). This instrument examines the emotional, cognitive, and behavioral characteristics of compulsive exercise from a multi-facet perspective. Further, the instrument also evaluates the extent to which the maintenance of compulsive exercise may be driven by the need to sustain a rigid schedule, to improve one’s mood despite a lack of enjoyment when exercising, or as a way to avoid negative emotions and feelings of guilt that may emerge when not exercising (Meyer et al., 2011; Taranis et al., 2011). The CET has been validated in athletes (Plateau et al., 2014), adolescents with ED (Formby et al., 2014; Noetel et al., 2016), adolescent community samples (Goodwin et al., 2011a, b), and in adults with AN (Young et al., 2017). However, there is still lacking a validation in a transdiagnostic adult sample with longstanding ED, and translations of the instrument need to be validated.

Compulsive exercise has been shown as a transdiagnostic symptom across various ED diagnoses, yet there are inconsistency with regards to actual differences across the diagnoses. Several studies have found that persons with AN show higher frequencies of exercise and higher prevalence of compulsive exercise compared to persons with BN, whereas others have found no differences across diagnoses (Pirke et al., 1991; Brewerton et al., 1995; Sundgot-Borgen et al., 1998; Solenberger, 2001; Penas-Lledo et al., 2002; Shroff et al., 2006). These results must also be interpreted in light of various assessment instruments and the aforementioned variations in conceptualization of compulsive exercise. One study found higher scores on compulsive exercise using the CET in persons with BN and EDNOS compared to AN (Sauchelli et al., 2016). Unfortunately, this study did not report ED duration in the clinical sample, hence it is difficult to evaluate if the sample can be generalized to persons with short term or longstanding, sustained ED.

One challenge with current understanding of compulsive exercise is the recently reported exercise paradox observed in persons with BN or BED (Mathisen et al., 2018). This exercise paradox refer to high scores on compulsive exercise instruments despite low levels of physical activity and absence of exercise behavior. One study (Bratland-Sanda et al., 2019) found 20% with BN and 6% of BED with high CET-score (i.e., ≥15), yet sedentary behavior (i.e., <150 min/week with physical activity). Individuals with ED tend to struggle with obsessiveness related to food, eating, shape and weight, and the compulsiveness of ED behaviors such as binge eating, restriction and/or compensating behavior (Altman and Shankman, 2009). It may therefore be phenomenological and syndromal overlap between ED and obsessive-compulsive disorder (OCD) when it comes to attitudes toward exercise. Therefore it is important to improve knowledge about both the obsessions regarding exercise, and the actual exercise performed. However, there are as far as our concern no studies that have investigated the diversity between obsession and compulsion of exercise in ED. The first aim of this study is to test the psychometric properties of the Norwegian version of CET among adults with longstanding ED. This aim is fourfold. First, to assess internal consistency of the CET. Second, to determine whether the previously reported factor structure of the CET is appropriate for use with a clinical group of longstanding ED. Third, to assess the convergent validity and fourth, to assess discriminative validity of the measure. The second aim is to explore predictors of high CET score.

## Materials and Methods

### Sample

The sample was inpatient with longstanding ED nested within a randomized controlled trial (RCT) from Modum Bad Psychiatric Center in Norway. A total of 166 adult patients – 163 female and 3 male - satisfying DSM-IV criteria for ED were consecutively recruited during period 2015–2017, receiving either cognitive-behavioral or compassion-focused inpatient therapy at a specialized ED unit at a psychiatric facility. In order to be eligible to participate in the RCT, the patients had to be at least 18-year old with an ED diagnosis: AN, BN, EDNOS, according to the Eating Disorder Examination-interview and provided informed consent. A potential participant who had current psychosis, serious substance abuse interfering with treatment or ongoing trauma (e.g., current involvement in an abusive relationship), were excluded from the RCT. A team of independent psychologists and psychiatrists with extensive training and experience in diagnostic assessment evaluated potential patients using standardized assessment tools.

Patients’ mean age was 35.9 years and mean BMI was 21.2 (Table 1). Forty-five individuals (27%) met criteria for AN, 68 (41%) for BN, and 53 (32%) for EDNOS. There were only significant differences between the diagnostic groups on the BMI (Table 1).

**TABLE 1:** Characteristics for all patients and the sample divided into diagnostic groups.

	**AN**	**BN**	**EDNOS**	**ANOVA**	**Total**
	***n* = 45**	***n* = 68**	***n* = 53**	***f*-value**	**(*N* = 166)**
BMI, (kg/m^2^)	17.2 (2.4)	23.2 (6.7)	22.0 (5.7)	19.6^∗∗∗^	21.21 (5.7)
Age, (yrs)	37.4 (11.8)	33.9 (9.1)	37.5 (12.7)	0.8	35.9 (11.0)
Duration of illness, (yrs)	19.0 (8.3)	19.7 (8.9)	19.8 (12.7)	0.0	19.5 (9.6)
Duration of treatment, (yrs)	8.2 (8.7)	5.8 (5.0)	6.9 (4.5)	0.6	6.4 (5.9)
CET					
Total	15.5 (4.5)	13.9 (4.4)	15.0 (4.1)	2.1	14.7 (4.4)
Avoidance and rule-driven behavior	3.0 (1.6)	2.4 (1.4)	2.8 (1.5)	2.3	2.7 (1.5)
Weight control	3.1 (1.1)	2.7 (1.1)	3.1 (1.2)	1.5	3.0(1.1.)
Mood improvement	3.8 (1.1)	3.6 (1.2)	3.8 (1.2)	0.7	3.7 (1.1)
Lack of exercise enjoyment	2.5 (0.7)	2.5 (0.6)	2.3 (0.6)	1.0	2.4 (0.6)
Exercise rigidity	3.1 (1.4)	2.6 (1.3)	2.9 (1.5)	1.6	2.9 (1.4)
EDE-Q					
Global	4.1 (1.2)	4.2 (1.2)	4.0 (1.3)	0.1	4.1 (1.2)
Restraint	4.1 (1.4)	3.7 (1.6)	3.8 (1.4)	0.4	3.9 (1.5)
Eating concern	3.3 (1.6)	3.6 (1.4)	3.3 (1.4)	0.5	3.5 (1.4)
Shape concern	4.7 (1.4)	5.0 (1.2)	4.8 (1.4)	0.3	4.9 (1.3)
Weight concern	4.1 (1.4)	4.3 (1.5)	4.2 (1.6)	0.3	4.2 (1.5)
BDI	30.5 (11.0)	30.3 (12.4)	29.9 (11.6)	0.0	30.2 (11.7)
SCL-90	1.6 (0.6)	1.6 (0.7)	1.5 (0.6)	0.4	1.6 (0.7)

*CET, Compulsive Exercise Test, EDE-Q, Eating Disorder examination questionnaire, BDI, Beck Depression Inventory, SCL-90, symptom checklist-90. ^∗∗∗^*p* < 0.0001.*

### Instruments

#### The Compulsive Exercise Test (Taranis et al., 2011)

The CET is a self-reported questionnaire designed to explore the emotional, cognitive and behavioral characteristics of compulsive exercise. It comprises 24 items answered on a 6-point Likert scale, from 0 (never true) to 5 (always true). CET consists of five subscales: “avoidance and rule-driven behavior,” “weight control exercise,” “mood improvement,” “lack of exercise enjoyment,” and “exercise rigidity.” Mean scores of each subscale are summarized to obtain a CET total score. The Cronbach’s α coefficient for CET for this study ranged from 0.68 to 0.96. One study found that a cut-off score of 15 on the CET resulted in acceptable values of both sensitivity and specificity to distinguish between clinical cases with and without compulsive exercise (Meyer et al., 2016). We used this cut-off to aid the identification of high CET score within our clinical population.

#### The Eating Disorder Examination, 16th Edition (EDE-I) (Fairburn et al., 2008)

Translated and validated in Norwegian (Reas et al., 2011). The EDE-I is an interviewer-administered measure with excellent psychometric properties (Luce and Crowther, 1999) that is widely used in the assessment of ED. Aside from generating ED diagnoses, the measure provides information on the frequency of ED behaviors (such as binge eating and purging). EDE-I was used in order to obtain ED diagnosis.

#### Eating Disorder Examination-Questionnaire Version 6.0 (Fairburn and Beglin, 1994)

Translated and validated in Norwegian (Rø et al., 2010). The EDE-Q is a self-report questionnaire adapted from the interview-based EDE and measures ED psychopathology. A mean value is calculated on a 0–6 point scale and the total score varies from 0 to 6. The EDE-Q consists of four subscales: “restraint,” “shape concern,” “weight concern,” and “eating concern.” A measure of Cronbach’s α in EDE-Q demonstrated good subscale reliability with α = 0.93 (global score), α = 0.79 (restriction), α = 0.74 (eating concern), α = 0.90 (shape concern) and α = 0.81 (weight concern).

#### Symptom Checklist-90 (Derogatis, 1983)

Translated and validated in Norwegian (Derogatis, 2010). The SCL-90 is one of the most widely used self-report scale of psychological distress in clinical practice and research. The measure consists of 90 items, and gives a GSI score of general distress. The items are rated on a Likert scale from 0 to 5, and the total GSI score varies from 0 to 5. SCL 90 has shown good psychometric properties (Schmitz et al., 2000). The Cronbach’s α coefficient for SCL-90 in this study was 0.97.

#### Beck Depression Inventory-II (BDI-II) (Beck et al., 1996)

Translated and validated in Norwegian (Nordhus et al., 2001). The BDI is a 21-item measure assessing level of depression. The items are scored on a Likert scale from 0 to 4 and the total range of scores is from 0 to 63. The psychometric properties of BDI are adequate (Beck et al., 1988). The Cronbach’s α coefficient for the BDI II for this study was 0.91.

#### Translation of the CET

The translation into Norwegian was conducted via the use of translation/back-translation. Two researchers in the field of ED, with Norwegian as native language and English skills at C2 level according to the Common European Framework of Reference for Languages (CEFR) (Council of Europe, 2001), provided a first translation from English to Norwegian. Thereafter, a psychiatrist in the field of ED, also with Norwegian as native language and English skills at C2 level according to the CEFR guidelines made a back-translation to English. Throughout the process, language, grammar, and cultural discrepancies that might influence the interpretation of the questionnaire items were taken into account.

### Statistical Analysis

IBM SPSS AMOS 25 was used for the statistical analyses. ANOVA was used to investigate group differences between the EDs. Cronbach’s α was used to assess internal consistency of CET. We conducted a confirmatory factor analysis to assess the fit of the CET five factor construct found in Taranis et al. (2011). The following goodness-of-fit indices were used: Root Mean Square Error Approximation (RMSEA) where value of <0.08 indicated good fit and values of 0.080–0.10 indicated mediocre fit (Kline, 2015), Comparative Fit Index (CFI) and Tucker Lewis Index (TLI) where values >0.90 indicated adequate fit (Kline, 2015) and Standardized Root Square Mean Residual (SRMR) where values <0.10 indicated adequate fit (Kline, 2015). Similar to Meyer et al. (2016), we defined factor loadings above 0.40 as appropriate (Ford et al., 1986). Validity was assessed by computing Pearson’s correlation coefficients for convergent validity between CET and EDE-Q, and discriminant validity between CET and BDI and SCL-90. Chi-square were used to test the difference between patients with high and low CET-score. Finally, to explore predictive contribution to high CET score, an enter logistic regression were conducted with sex, age, global EDE-Q, frequencies of exercise (question 18 in EDE-Q) and ED diagnosis as independent variables.

In this study, results were considered statistically significant at a significance level of *p* < 0.05. Since *p*-values depend on both the magnitude of associations and the precision of the estimate; both the significance test and estimation of effect sizes (Cohen’s d) were taken into account.

## Results

### Validation of the Norwegian Version of CET

#### Internal Consistency

A measure of internal consistency (Cronbach’s α) demonstrated good subscale reliability for the model of CET, except for the CET subscale weight control (Table 2).

**TABLE 2:** Confirmatory factor analysis for the 5-factor model of CET and Cronbach’s α for the subscales, *N* = 166.

**Items**	**B**	**SE**	***p*-**	**CI**	**CI**
			**value**	**Lower**	**Upper**
**Avoidance (α = 0.96)**					
CET9 If I cannot exercise I feel low or depressed	0.918	0.018	0.023^∗^	0.866	0.949
CET10 I feel extremely guilty if I miss an exercise session.	0.908	0.022	0.023^∗^	0.842	0.947
CET11 I usually continue to exercise despite injury or illness, unless I am very ill or too injured	0.759	0.043	0.021^∗^	0.624	0.829
CET15 If I miss an exercise session, I will try and make up for it when I next exercise	0.775	0.042	0.007^∗∗^	0.687	0.857
CET16 If I cannot exercise I feel agitated and/or irritable	0.907	0.018	0.013^∗^	0.867	0.940
CET20 If I cannot exercise I feel angry and/or frustrated	0.907	0.019	0.018^∗^	0.861	0.936
CET22 I feel like I’ve let myself down if I miss an exercise session	0.820	0.044	0.023^∗^	0.705	0.888
CET23 If I cannot exercise I feel anxious	0.902	0.021	0.015^∗^	0.852	0.934
**Weight control (α = 0.68)**					
CET2 I exercise to improve my appearance	0.670	0.068	0.021^∗^	0.471	0.771
CET6 If I feel I have eaten too much, I will do more exercise	0.827	0.043	0.015^∗^	0.727	0.898
CET8 I do not exercise to be slim	−0.192	0.107	0.058	–0.460	0.007
CET13 I exercise to burn calories and lose weight	0.810	0.048	0.014^∗^	0.698	0.882
CET18 I cannot exercise, I worry that I will gain weight	0.897	0.029	0.019^∗^	0.816	0.939
**Mood improvement (α = 0.92)**					
CET1 I feel happier and/or more positive after I exercise	0.859	0.033	0.009^∗∗^	0.779	0.914
CET4 I feel less anxious after I exercise.	0.707	0.059	0.015^∗^	0.553	0.810
CET14 I feel less stressed and/or tense after I exercise	0.896	0.023	0.023^∗^	0.843	0.932
CET17 Exercise improves my mood	0.863	0.049	0.019^∗^	0.721	0.923
CET24 I feel less depressed or low after I exercise	0.884	0.039	0.014^∗^	0.787	0.947
**Lack of enjoyment (α = 0.88)**					
CET5 I find exercise a chore.	0.711	0.081	0.007^∗∗^	0.509	0.845
CET12 I enjoy exercising.	−0.869	0.083	0.007^∗∗^	–1.087	–0.767
CET21 I do not enjoy exercising.	0.717	0.110	0.018^∗^	0.426	0.880
**Exercise rigidity**					
CET3 I like my days to be organized and structured of which exercise is just one part	0.658	0.059	0.006^∗∗^	0.544	0.759
CET7 My weekly pattern of exercise is repetitive.	0.897	0.033	0.012^∗^	0.821	0.952
CET19 I follow a set routine for my exercise sessions e.g., walk or run the same route, particular exercises, same amount of time, and so on.	0.806	0.046	0.016^∗^	0.691	0.885

*CET, Compulsive Exercise Test. ^∗^*p* < 0.01, ^∗∗^*p* < 0.001, ^∗∗∗^*p* < 0.0001.*

#### Confirmatory Factor Analysis

The five-dimension structure showed adequate fit [χ^2^ (242) = 525.07, *p* < 0.001, RMSEA = 0.084 (90%CI 0.074–0.094], CFI = 0.906, TLI = 0.884, SRMR = 0.086). Factor loadings were appropriate for all items except Item 8 (CET Weight control). All CET subscales were correlated with each other except for the CET Lack of enjoyment (Table 2). When replicated the confirmatory factor analysis in the diagnostic subgroups, the factor loading did not change.

#### Convergent Validity

Convergent validity of the CET was assessed by correlation analyses with EDE-Q (Table 3). The EDE-Q global score was correlated with CET total score, CET Avoidance and rule-driven behavior, CET Weight control, and CET Lack of enjoyment (Table 3). The same pattern was found for correlation between CET subscales and EDE-Q subscales.

**TABLE 3:** Convergent validity between the CET and EDE-Q, *N* = 166.

**Measures**	**1**	**2**	**3**	**4**	**5**	**6**	**7**	**8**	**9**	**10**	**11**
(1) CET Total	–	0.89^∗∗∗^	0.84^∗∗∗^	0.67^∗∗∗^	0.25^∗∗^	0.85^∗∗∗^	0.33^∗∗∗^	0.33^∗∗∗^	0.26^∗∗^	0.28^∗∗∗^	0.27^∗∗∗^
(2) CET Avoidance and rule-driven behavior		–	0.76^∗∗∗^	0.43^∗∗∗^	0.07	0.73^∗∗∗^	0.39^∗∗∗^	0.37^∗∗∗^	0.31^∗∗∗^	0.33^∗∗∗^	0.36^∗∗∗^
(3) CET Weight control			–	0.42^∗∗∗^	0.18^∗^	0.58^∗∗∗^	0.50^∗∗∗^	0.43^∗∗∗^	0.40^∗∗∗^	0.47^∗∗∗^	0.43^∗∗∗^
(4) CET Mood improvement				–	0.09	0.44^∗∗∗^	–0.02	0.03	–0.03	–0.02	–0.06
(5) CET Lack of exercise enjoyment					–	0.07	0.19^∗^	0.13	0.22^∗∗^	0.13	0.17^∗^
(6) CET Exercise rigidity						–	0.14	0.20^∗∗^	0.10	0.10	0.08
(7) EDE-Q total							–	0.82^∗∗∗^	0.82^∗∗∗^	0.90^∗∗∗^	0.91^∗∗∗^
(8) EDE-Q Restraint								–	0.57^∗∗∗^	0.62^∗∗∗^	0.65^∗∗∗^
(9) EDE-Q Eating concern									–	0.63^∗∗∗^	0.64^∗∗∗^
(10) EDE-Q Weight concern										–	0.87^∗∗∗^
(11) EDE-Q Shape concern											–

*CET, Compulsive Exercise Test; EDE-Q, Eating Disorder examination-questionnaire. ^∗^*p* < 0.01, ^∗∗^*p* < 0.001, ^∗∗∗^*p* < 0.0001.*

#### Discriminant Validity

Table 4 shows the correlations between the CET and BDI and SCL-90. There were significant correlations between the CET Total, CET Avoidance and rule-driven behavior, CET Weight control and BDI and between CET Avoidance and rule-driven behavior and SCL-90. Otherwise there were no significant correlations.

**TABLE 4:** Discriminant validity between the CET and BDI and SCL-90, *N* = 166.

**Measures**	**BDI**	***p***	**SCL-90**	***p***
CET Total	0.19^∗^	0.02	0.14	0.08
CET Avoidance and rule-driven behavior	0.32^∗∗∗^	0.001	0.30^∗∗^	0.001
CET Weight control	0.25^∗∗^	0.002	0.15	0.06
CET Mood improvement	–0.11	0.16	–0.10	0.21
CET Lack of exercise enjoyment	0.16	0.06	0.03	0.71
CET Exercise rigidity	0.07	0.37	0.06	0.46

*CET, Compulsive Exercise test; BDI, Beck Depression Inventory; SCL-90, symptom checklist-90. ^∗^*p* < 0.01, ^∗∗^*p* < 0.001, ^∗∗∗^*p* < 0.0001.*

### Prediction of High CET Score

By using the CET cut off ≥15 based on Meyer et al. (2016), we found that 80 patients (52%) had a high CET score. The distribution of high CET score across ED diagnoses were 67% in AN, 43% in BN and 52% in EDNOS [χ^2^ (2) = 5.69, *p* = 0.06]. A logistic regression analysis was conducted to assess predictors of the high CET scores, and this showed that the total variance in high CET scores that could be explained by the frequency of exercise and the EDE global score was 33% (Table 5). Sex, age and ED diagnosis did not predict high CET score.

**TABLE 5:** Logistic regression analysis for prediction of high CET score, *N* = 166.

	**B**	**SE**	***R*^2^**	***p***	**Exp B**	**95%CI**	**95%CI**
						**low**	**upper**
**Step 1**							
Episodes of exercise	0.122	0.021	0.30	<0.001	1.130	1.083	1.178
**Step 2**							
Episodes of exercise	0.108	0.022	0.32	<0.001	1.114	1.068	1.163
EDE-Q global score	0.419	0.196		0.033	1.521	1.035	2.236

*Variables excluded from the model: gender, ED diagnosis, age. EDE-Q, Eating Disorder examination-questionnaire.*

**FIGURE 1:**
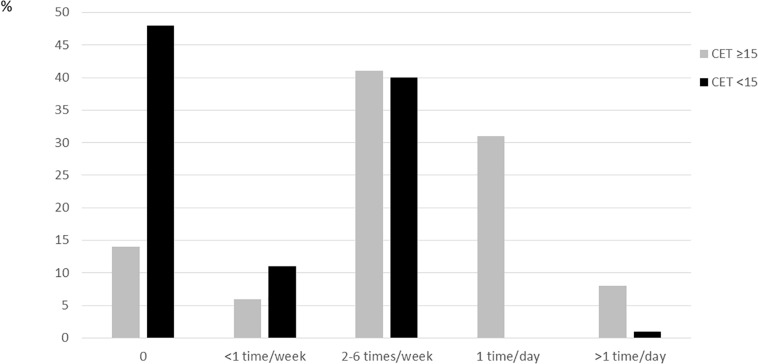
Proportion of subjects with high (≥15) or low (<15) CET-score across frequency of exercise, from 0 to >1 time/day.

When examining frequency of exercise, 39% of patients with high CET score and 1% of patients with low CET score reported daily episodes with exercise (Figure 1). Among the patients with high CET score, 20% reported less than one episode of exercise per week the past 28 days.

## Discussion

The aim of this study was to test the psychometric properties of the Norwegian version of CET, and examine predictors of high CET score, among adults with longstanding ED.

The confirmatory factor analysis revealed that the clinical data showed an adequate goodness-of-fit to the previously published five factor model (Goodwin et al., 2011a; Taranis et al., 2011), providing further support for the multidimensional nature of compulsive exercise (Meyer et al., 2011). Similar to Meyer et al. (2011) we found poor fit of one items (8), but neither them nor we found removal of this item to change the fit of the five-factor structure. Since the factor loadings for this item remain low in this replication, it might be advisable to remove this from the scale. However, further research is required to eventually replicate this findings, specifically invariant testing across control and clinical samples and with clinical samples including men.

The measure demonstrated good internal consistency, convergent and discriminant validity. However, Mood improvement did not correlate with any EDE subscales. Previous research has shown that the Mood improvement subscale is not strongly associated with eating psychopathology among several different groups of patients (Goodwin et al., 2011a; Taranis et al., 2011; Plateau et al., 2014; Sauchelli et al., 2016). When determining the severity of compulsive exercise within each person, results from this subscale should be interpreted with caution. It should also be kept in mind that Avoidance and rule-driven behavior correlated with both BDI and SCL-90. This may be explained by the fact that one of the nine primary symptom dimensions in SCL-90 is Obsessive-compulsiveness. This can count for a conceptual overlap between rule-driven behavior measured by CET and obsession measured by SCL-90.

In contrast to Sauchelli et al. (2016), we did not detect differences in CET score among patients with different ED diagnosis. This may be explained by age differences between the two samples, and possible differences in ED duration. It may also be due to a type II error in our sample since the *p*-value was close to the significance level. Nevertheless, this finding show that compulsive exercise is prevalent in individuals across diagnoses AN, BN, and EDNOS, however this may only hold for longstanding ED. The reported factor structure was consistent across diagnostic groups, confirming the equality between different ED diagnoses.

Some additional findings were noted in the current study. Higher frequency of episodes of exercise predicted high CET score, however 21% of the patients with high CET score had no or little actual exercise behavior. This indicates presence of a subtype within compulsive exercise showing high loading on exercise obsessions, yet without actually performing exercise. As a result, the current finding suggest that an understanding of compulsive exercise must to a greater extent differ between obsessions and compulsions. Some studies hypothesize that some common phenotype characteristics are shared by AN and OCD (Halmi et al., 2003) and in one study of OCD symptoms in women with BN, 39% of the subjects were found to have obsessions related to symmetry and exactness (Von Ranson et al., 1999). It may be that some of these obsessions are largely egosyntonic (Mazure et al., 1994) and in that sense leads to compulsive and ritualistic behavior. Trying to understand exercise obsessions without compulsive behavior in persons with ED, two explanations are possible. On one hand, such obsessions can be understood as egodystonic and unwanted, and that they restrict themselves from performing exercise and thus this may lead to more negative affect. On the other hand, exercise is an important factor for public health, and it is something that is encouraged to integrate into daily life throughout the life course. In the general population, there is also a challenge with sedentary behavior despite knowledge of the benefits of exercise, and the discrepancy between intentions to exercise and actual behavior can arise shame, guilt and lack of self-efficacy (Streuber et al., 2015). Thus, when the exercise obsessions are involuntarily restricted they can negatively reinforce the experienced guilt and shame regarding the sedentary behavior (Flora et al., 2012).

### Strength and Limitations

This is the first study to validate a Norwegian translation of the CET, and hence it provides the groundwork for further research into this multi-faceted perspective of compulsive exercise. The inclusion of a large inpatient sample with comparison across ED diagnostic subtypes also adds strength to the study. Although the findings above supported the reliability, validity, and clinical utility of the CET, limitations should be noted. The assessment of compulsive exercise was made via the use of a participative measure, which is vulnerable to reporting bias. Future studies should consider the use of objective instruments to assess the actual amount of exercise (i.e., frequency, intensity, and duration). We acknowledge there are other valid and reliable objective exercise measures, such as accelerometers. But measures were constrained to those selected for the RCT and to limit potential participant fatigue. Our sample was patients with longstanding ED receiving inpatient treatment. Because of the symptom severity one may argue that the patients in the study are selected, and preventing the results to be generalized to patients with ED in general as the present series represents an atypical subset of patients. Thus, these results may not generalize to people with ED receiving outpatient treatment and are medical stable. However, all diagnostic categories were represented, reflecting the diagnostic distribution commonly seen in clinical practice. Males were underrepresented and clearly, the utility of the measure relies upon the factor structure being stable with ED for both genders.

### Clinical Implications

Assessing compulsive exercise should be an important component of treatment programs. Our study shows the importance of not only assess the actual behavior, but also the very ED preoccupations that can contribute to the onset and maintenance of ED. Identification and management of exercise obsessions should be addressed in treatment settings, and it is important to gain more knowledge on the function and possible consequences of exercise obsessions with absence of actual exercise behavior.

## Conclusion

In conclusion, the Norwegian version of CET is valid and useful for assessing compulsive exercise in adults admitted to inpatient treatment for longstanding ED. ED severity and exercise behavior predict high CET score, although a subgroup of persons with high CET score only show presence of exercise obsessions.

## Data Availability Statement

The datasets generated and analyzed during the current study are not publicly available due to Norwegian laws and regulations, but are available from the corresponding author on reasonable request.

## Ethics Statement

The study was approved by the South-Eastern Regional Committee for Medical and Health Research Ethics of Norway (REC approval; 2014/836) and Clinical trials: NCT02649114. All patients gave their written consent to participation.

## Author Contributions

KV made substantial contributions to the conception and design, the data collection, analysis and interpretation of data, and drafting and revising of the manuscript. SB-S made substantial contributions to the conception and design, the analysis and interpretation of data, and drafting and revising of the manuscript. Both authors have approved the final version of the manuscript, and agreed to be accountable for all aspects of the work in ensuring that questions related to the accuracy or integrity of any part of the work are appropriately investigated and resolved.

## Conflict of Interest

The authors declare that the research was conducted in the absence of any commercial or financial relationships that could be construed as a potential conflict of interest.

## Abbreviations

AN: anorexia nervosa
BDI-II: Beck Depression Inventory nb 2
BED: Binge Eating Disorder
BMI: body mass index
BN: bulimia nervosa
DSM-IV: Diagnostic and Statistical Manual nb. IV
ED: eating disorder
EDE-Q: eating disorder examination-questionnaire
EDNOS: Eating Disorder Not Otherwise Specified
SCL-90: symptom checklist-90.
